# Effect of zooming on texture features of ultrasonic images

**DOI:** 10.1186/1476-7120-4-8

**Published:** 2006-01-28

**Authors:** Stavros K Kakkos, Andrew N Nicolaides, Efthyvoulos Kyriacou, Constantinos S Pattichis, George Geroulakos

**Affiliations:** 1 Department of Vascular Surgery, Imperial College, London, UK; 2 The Cyprus Institute of Neurology and Genetics, Nicosia, Cyprus; 3 Department of Computer Science, University of Cyprus, Nicosia, Cyprus; 4 Department of Vascular Surgery, Ealing Hospital, London, UK

## Abstract

**Background:**

Unstable carotid plaques on subjective, visual, assessment using B-mode ultrasound scanning appear as echolucent and heterogeneous. Although previous studies on computer assisted plaque characterisation have standardised B-mode images for brightness, improving the objective assessment of echolucency, little progress has been made towards standardisation of texture analysis methods, which assess plaque heterogeneity. The aim of the present study was to investigate the influence of image zooming during ultrasound scanning on textural features and to test whether or not resolution standardisation decreases the variability introduced.

**Methods:**

Eighteen still B-mode images of carotid plaques were zoomed during carotid scanning (zoom factor 1.3) and both images were transferred to a PC and normalised. Using bilinear and bicubic interpolation, the original images were interpolated in a process of simulating off-line zoom using the same interpolation factor. With the aid of the colour-coded image, carotid plaques of the original, zoomed and two resampled images for each case were outlined and histogram, first order and second order statistics were subsequently calculated.

**Results:**

Most second order statistics (21/25, 84%) were significantly (p < 0.05) sensitive to image zooming during scanning, in contrast to histogram and first order statistics (4/25, 16%, p < 0.001, Fisher's exact test). Median (interquartile range) change of those features sensitive to zooming was 18.14% (4.94–28.43). Image interpolation restored these changes, the bicubic interpolation being superior compared to bilinear interpolation (p = 0.036).

**Conclusion:**

Texture analysis of ultrasonic plaques should be performed under standardised resolution settings; otherwise a resolution normalisation algorithm should be applied.

## Background

Cross-sectional studies have shown that echolucent and heterogeneous internal carotid artery plaques on B-mode ultrasound scanning are associated with neurological symptoms [1-3]; similarly prospective studies have confirmed that these subjective plaque characteristics predict future neurological symptoms [4,5]. Our group has investigated objective, computer-assisted methods, which involved standardisation of ultrasonic images (normalisation) and echogenicity measurements [6,7]. We have also, like other groups, investigated objective methods of accessing plaque heterogeneity, known also as texture analysis, and found these helpful in separating symptomatic from asymptomatic plaques [8-12].

Image resolution has a significant effect on texture analysis results; this has been shown by studies on remote sensing [13-15], and ultrasound [16]. Images obtained during ultrasound scanning can have variable resolution due to different zooming (resampling) factors during the actual scanning procedure and digitisation settings during image downloading. Kuo, in an effort to solve this problem, proposed an algorithm, which ignores the extra pixels of those images with increased resolution [17]. The aim of the present study was to investigate the influence of image zooming during ultrasound scanning on the value of histogram analysis and textural features and to test whether or not resolution standardisation by applying image resampling decreases the variability introduced by the different image resolution.

## Methods

**Figure 1:**
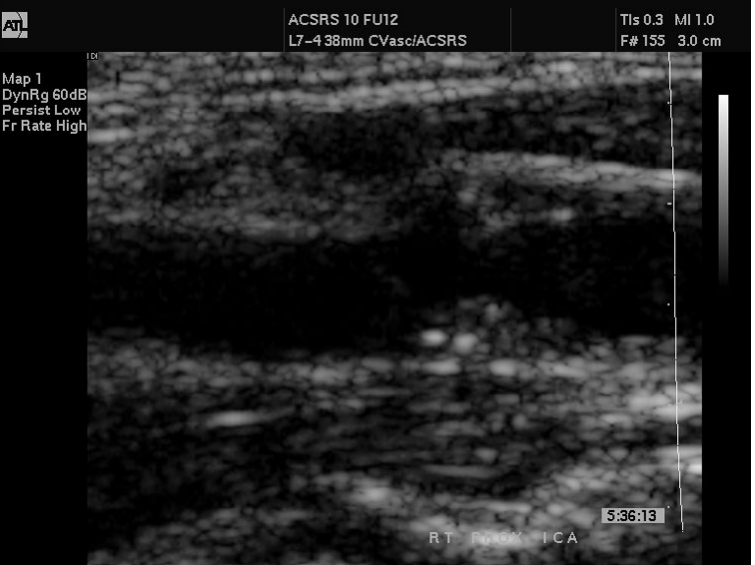
This figure shows an unzoomed B-mode still image obtained during carotid scanning.

**Figure 2:**
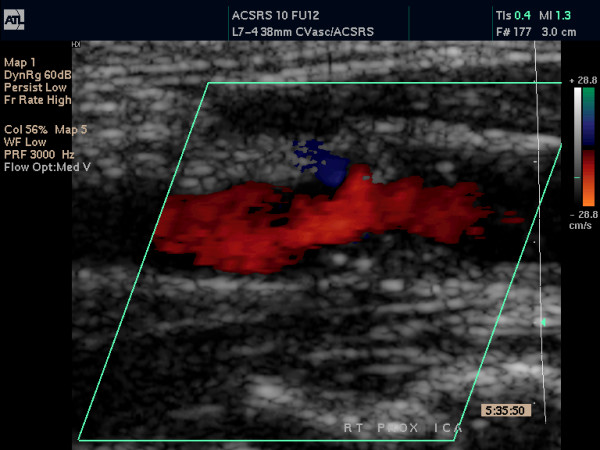
This figure shows the unzoomed colour-coded still image, corresponding to Figure 1. Image was used to facilitate the outline of the original gray scale plaque shown in Figure 1, during image analysis.

**Figure 3:**
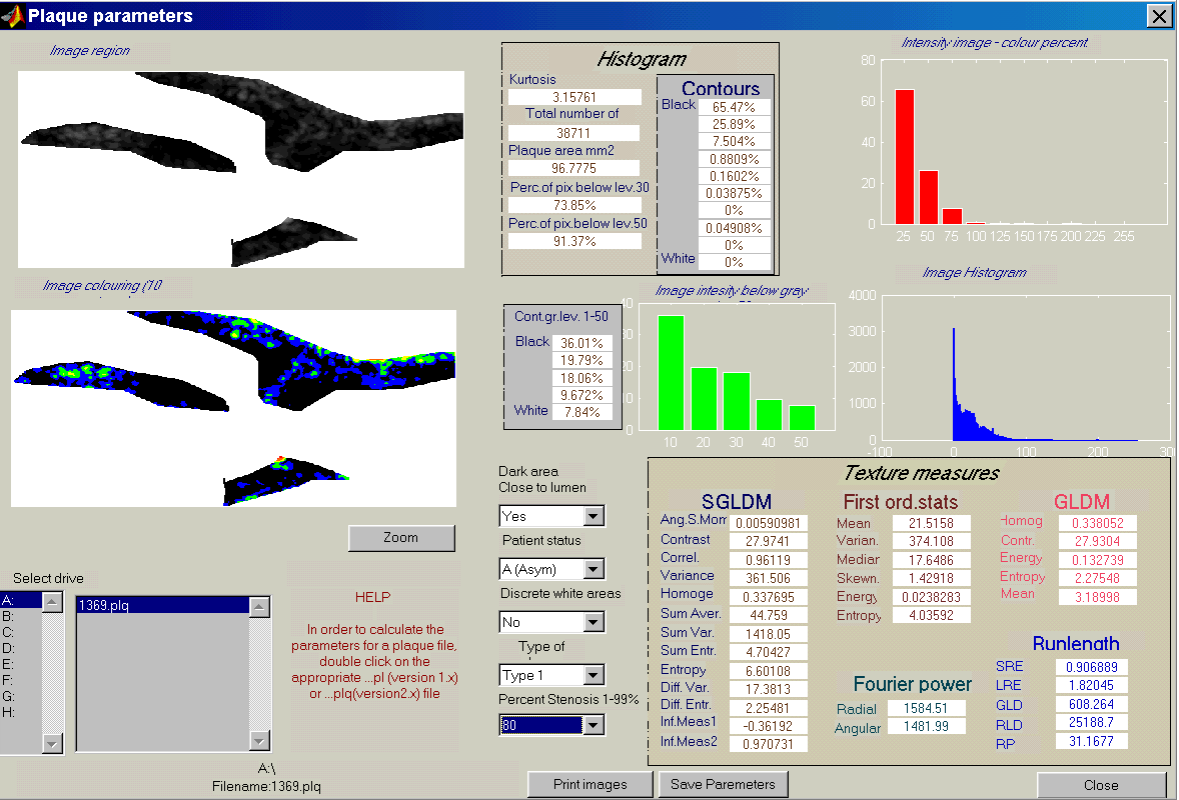
Histogram and statistical features of the carotid plaque outline (top left), were automatically extracted by the computer program module, shown in this figure. The contoured image (10 contours of the 0–255 grey level spectrum) is shown below.

Eighteen images of carotid plaques producing stenosis greater than 50% were included in this study. These were obtained from consecutive asymptomatic patients, participants of the Asymptomatic Carotid Stenosis and Risk of Stroke (ACSRS) multicenter natural history study [18]. Stenosis severity was estimated with velocity ratios (European Carotid Surgery Trial – ECST – method), as previously described [19], using an ATL HDI 3000 scanner (Philips Medical Systems, Bothell, WA, USA). A linear post-processing curve was used during carotid scanning, B-mode and colour-coded still images (Figures 1 and 2) were stored on magneto-optical disks as Tagged Image File Format (TIFF) files [resolution of 576 pixels (height) × 768 pixels (width)]; the same still (frozen) B-mode images were zoomed off-line using the zoom feature of the scanner (zoom factor of 1.3, default of the ultrasound scanner) and also stored on the magneto-optical disk. B-mode images were 8-bit i.e. they had 256 (range 0–255) shades of grey. All digital (unzoomed and zoomed) images were recorded using a standardised protocol [7,10] and subsequently transferred to a PC and normalised for brightness, using blood and arterial adventitia as reference points, as previously described [7], using commercially available software (Adobe ^® ^Photoshop version 5.5, Adobe Systems Inc., Palo Alto, CA, USA). Normalisation (linear scaling) of the image was performed with the "curves" option of the software so that in the secondary image the grey scale median (GSM) of blood is 0 to 5 and that of the adventitia is 185 to 195. To reduce variability, a single GSM measurement of reference points (adventitia and blood) was used for the process of normalisation of both the unzoomed and zoomed images. Subsequently, the normalised resolution, i.e. the number of pixels per cm of image depth (using the image depth scale) was calculated. Although in some of the images, due to deeply situated carotid arteries, image depth was increased and therefore normalised resolution decreased, it was realised that the zoomed image had invariably increased resolution, 1.3 times more than the unzoomed image. The original B-mode images were subsequently interpolated (resampled) to increase their *pixel *resolution 1.3 times, to match the zoom factor of the scanner and therefore simulate the zoom process of the scanner. This resolution standardisation was achieved by using the image size (resampling) feature of the Adobe ^® ^Photoshop software (version 5.5). The bilinear and bicubic interpolation methods (Appendix I) were used to resample the images. With the aid of the colour-coded image, the region of interest (carotid plaques) of the original, the zoomed and two resampled images (all grey-scale or B-mode) for each case were outlined and texture features were calculated. Texture analysis of the plaque outlines was performed with a custom-made computer program (Figure 3) and a MATLAB platform (The MathWorks, Inc, Natick, Mass, USA); the program also counts the number of pixels included in the plaque outline. Results were saved by downloading them to a text file, which can be imported by most statistical packages. Textural features calculated included:

A. Histogram measures

Percentage of pixels below grey level 30 (PP < 30) and 50 (PP <50).

Percentage of pixels of each of the 10 contours of the 0–255 grey level spectrum (PPC1-PPC10), the first 2 contours (grey level 0–51) analysed further into 5 sub-contours (PPCS1-PPCS5). These are novel features described by the authors. PPC1 is the percentage of image pixels having a grey level between 0–26, PPCS1 is the percentage of image pixels having a grey level between 0–10, ect.

B. First order grey level parameters [20,21]

Mean grey level, variance, median (GSM), mode, kurtosis, skewness, energy, entropy.

C. Second order (texture) statistics

1. The Spatial Gray Level Dependence Matrices (SGLDM) algorithm, known also as co-occurrence matrix method [22]. We used an interpixel distance (d) of 1 and an average angle measure calculated by averaging the values from the measures calculated at angles 0, 45, 90 and 135, as previously described [12,23,24]. The following features were calculated: angular second moment (ASM), contrast, correlation, variance (sum of squares), inverse difference moment (IDM), sum average, sum variance, sum entropy, entropy, difference variance, difference entropy, information measures of correlation-1 and -2 (InM1 and InM2).

2. Gray level difference statistics (GLDS) [25]: Entropy, contrast, mean, angular second moment – Homogeneity, energy.

3. Gray level run length statistics [26]: Short run emphasis (SRE), grey level distribution (GLD), run length distribution (RLD), long run emphasis (LRE), run percentage (RP).

4. Radial and angular sum of the Fourier power spectrum (FPS) were calculated [25].

### Statistics

Because of the small sample size (<50), the Shapiro-Wilk test was used to test the results for normal distribution; because some of them were not normally distributed, the Wilcoxon signed ranks test was used to test the difference between unzoomed and zoomed images.

The results were expressed as median and interquartile range (IQR). SPSS for Windows, version 11.5 (SPSS Inc., Chicago, IL, USA), was the statistical package used for statistical analysis. P values of 0.05 or less were considered statistically significant.

## Results

Image zooming increased both plaque total pixel number and image resolution. Median (IQR) pixel count of unzoomed images was 9,629 (7,203–14,299). This was increased by 54.3% (~1.3^2 ^times) to 14,861 (11,595–22,673) with image zooming (p < 0.001). Median resolution of the original images used in the current study was 15.8 pixels/mm, which increased up to 20.55 pixels/mm with zooming (~1.3 times).

The results of texture analysis of the original, zoomed and resampled images are shown in Table 1, 2, 3, 4, 5, 6, 7. Twenty-five features (50%) were sensitive to zooming, and in five of them (10%), the magnitude of change was over 50%. Median (IQR) change of those features sensitive to zooming was 18.14% (4.94–28.43). Histogram features (Tables 1 and 2) and first order statistics (Table 3) were generally not sensitive to resolution changes, with only four of them being significantly (p < 0.05) sensitive (4/25, 16%).

**Table 1:** Table showing the difference of texture features (contour analysis) in zoomed images and those without zoom. The latter were subsequently resampled to equalise their resolution (pixels/mm) with the corresponding zoomed ones, by using bilinear (BLI) or bicubic (BCI) interpolation. Percent difference with zoomed image are also shown. Statistically significant differences are highlighted.

**Histogram features**	**Image group**	**Feature value (median, interquartile range)**	**Difference with original (x1) image %**	**p**
PPC1	Zoomed	37.53, 26.67–55.60		
	Unzoomed	34.89, 24.33–54.30	-7.04	0.094
	BLI	32.56, 25.02–51.56	-13.24	0.286
	BLC	37.68, 22.87–56.12	0.41	0.948
PPC2	Zoomed	24.32, 19.87–29.12		
	Unzoomed	25.03, 19.51–28.74	2.90	0.616
	BLI	25.37, 18.90–30.84	4.31	0.616
	BLC	25.19, 20.28–28.80	3.59	0.528
PPC3	Zoomed	14.87, 11.07–19.77		
	Unzoomed	16.98, 11.37–20.84	14.16	0.071
	BLI	16.93, 11.75–20.01	13.82	0.267
	BLC	16.08, 10.64–20.15	8.09	0.983
PPC4	Zoomed	7.86, 6.33–13.21		
	Unzoomed	8.53, 6.56–13.28	8.48	0.157
	BLI	8.22, 6.11–12.83	4.56	0.5
	BLC	7.56, 5.61–13.57	-3.84	0.231
PPC5	Zoomed	4.30, 2.93–8.25		
	Unzoomed	4.24, 3.60–7.18	-1.32	0.372
	BLI	4.21, 2.56–7.39	-2.08	0.879
	BLC	4.46, 2.28–7.34	3.90	0.372
PPC6	Zoomed	2.45, 0.92–5.35		
	Unzoomed	2.19, 1.41–5.45	-10.65	0.616
	BLI	2.39, 0.71–5.09	-2.26	0.586
	BLC	2.30, 0.78–4.48	-5.86	0.102
PPC7	Zoomed	1.15, 0.16–2.91		
	Unzoomed	1.11, 0.36–2.53	-3.73	0.687
	BLI	1.00, 0.29–2.55	-13.10	0.266
	BLC	1.04, 0.18–2.37	-9.46	0.586
PPC8	Zoomed	0.356, 0.000–1.341		
	Unzoomed	0.494, 0.000–1.427	38.94	0.638
	BLI	0.366, 0.009–1.435	2.77	0.47
	BLC	0.346, 0.000–1.284	-2.77	0.638
PPC9	Zoomed	0.007, 0.000–0.632		
	Unzoomed	0.043, 0.000–0.630	526.85	0.79
	BLI	0.003, 0.000–0.622	-61.12	0.441
	BLC	0.010, 0.000–0.611	43.62	0.333
PPC10	Zoomed	0, 0–0		
	Unzoomed	0, 0–0	N/A	0.465
	BLI	0, 0–0	N/A	0.715
	BLC	0, 0–0	N/A	0.273

**Table 2:** Table showing the difference of histogram features (subcontour analysis), PP < 30 and PP < 50 in zoomed images and those without zoom. The latter were subsequently resampled to equalise their resolution (pixels/mm) with the corresponding zoomed ones, by using bilinear (BLI) or bicubic (BCI) interpolation. Percent difference with zoomed image are also shown. Statistically significant differences are highlighted.

**Histogram features**	**Magnification**	**Feature value (median, interquartile range)**	**Difference with original (x1) image %**	**p**
PPCS1	Zoomed	14.56, 9.63–29.23		
	Unzoomed	14.09, 9.07–29.07	-3.22	0.679
	BLI	13.26, 9.98–24.53	-8.97	0.811
	BLC	17.36, 10.52–29.46	19.24	0.215
PPCS2	Zoomed	12.84, 8.96–18.60		
	Unzoomed	13.07, 7.91–17.01	1.84	**0.022**
	BLI	13.04, 8.93–16.14	1.56	0.286
	BLC	13.48, 7.66–16.28	5.03	0.17
PPCS3	Zoomed	12.24, 9.54–14.75		
	Unzoomed	11.58, 9.26–14.06	-5.38	**0.012**
	BLI	11.43, 9.45–13.81	-6.59	**0.031**
	BLC	12.32, 8.62–14.75	0.67	0.215
PPCS4	Zoomed	10.10, 7.97–11.71		
	Unzoomed	11.02, 7.93–13.52	9.15	0.396
	BLI	11.00, 7.74–13.29	8.88	0.472
	BLC	10.76, 8.64–12.90	6.52	0.248
PPCS5	Zoomed	8.14, 6.27–11.00		
	Unzoomed	8.45, 6.17–11.01	3.77	0.879
	BLI	8.40, 7.00–10.98	3.21	0.396
	BLC	7.59, 6.74–11.08	-6.79	0.879
PP < 30	Zoomed	44.10, 32.33–59.75		
	Unzoomed	42.43, 29.35–58.38	-3.78	0.071
	BLI	38.91, 30.26–57.52	-11.77	0.248
	BLC	43.82, 27.97–61.06	-0.64	0.948
PP < 50	Zoomed	67.20, 51.71–75.61		
	Unzoomed	66.06, 50.39–74.18	-1.69	0.071
	BLI	66.23, 52.45–75.02	-1.44	0.306
	BLC	67.22, 50.10–76.80	0.03	0.5

**Table 3:** Table showing the difference of first order statistics in zoomed images and those without zoom. The latter were subsequently resampled to equalise their resolution (pixels/mm) with the corresponding zoomed ones, by using bilinear (BLI) or bicubic (BCI) interpolation. Percent difference with zoomed image are also shown. Statistically significant differences are highlighted.

**First order statistic**	**Magnification**	**Feature value (median, interquartile range)**	**Difference with original (x1) image %**	**p**
Mean value	Zoomed	43.67, 33.23–59.62		
	Unzoomed	44.60, 34.06–60.07	2.12	0.145
	BLI	45.43, 33.98–58.29	4.03	0.5
	BLC	43.32, 33.51–58.38	-0.80	0.472
Variance	Zoomed	1,287.35, 800.78–2,083.38		
	Unzoomed	1,294.56, 1,054.95–2,111.79	0.56	0.349
	BLI	1,334.25, 831.38–2,082.33	3.64	0.616
	BLC	1,264.28, 852.92–2,058.30	-1.79	0.396
Median value	Zoomed	34.35, 20.73–48.31		
	Unzoomed	37.41, 22.67–49.98	8.90	**0.043**
	BLI	38.65, 24.26–48.22	12.51	0.349
	BLC	35.09, 21.55–50.40	2.16	0.913
Mode	Zoomed	8.00, 0.00–40.25		
	Unzoomed	3.50, 0.00–36.75	-56.25	0.341
	BLI	2.00, 0.00–31.00	-75.00	0.282
	BLC	1.00, 0.00–23.25	-87.50	0.137
Skewness	Zoomed	1.247, 0.728–1.520		
	Unzoomed	1.201, 0.827–1.478	-3.72	0.616
	BLI	1.188, 0.847–1.460	-4.76	0.879
	BLC	1.240, 0.812–1.484	-0.58	0.913
Energy	Zoomed	0.014, 0.011–0.027		
	Unzoomed	0.014, 0.011–0.024	-2.23	0.811
	BLI	0.0109, 0.0084–0.0173	-22.13	**<0.001**
	BLC	0.0115, 0.0089–0.0214	-17.45	**0.016**
Entropy	Zoomed	4.51, 4.15–4.71		
	Unzoomed	4.52, 4.19–4.72	0.33	0.879
	BLI	4.70, 4.48–4.98	4.24	**<0.001**
	BLC	4.68, 4.36–4.87	3.80	**<0.001**
Kurtosis	Zoomed	2.06, 1.71–2.49		
	Unzoomed	2.15, 1.76–2.47	4.49	**0.025**
	BLI	1.73, 1.71–1.81	-15.72	**0.006**
	BLC	1.76, 1.72–1.95	-14.37	**0.01**

**Table 4:** Table showing the difference of SGLDM features in zoomed images and those without zoom. The latter were subsequently resampled to equalise their resolution (pixels/cm) with the corresponding zoomed ones, by using bilinear (BLI) or bicubic (BCI) interpolation. Percent difference with zoomed image are also shown. Statistically significant differences are highlighted.

**SGLDM feature**	**Magnification**	**Feature value (median, interquartile range)**	**Difference with original (x1) image %**	**p**
ASM#	Zoomed	0.00148, 0.00069–0.00611		
	Unzoomed	0.00111, 0.00060–0.00359	-24.61	**0.016**
	BLI	0.00097, 0.00059–0.00323	-34.05	**0.001**
	BLC	0.00108, 0.00056–0.00902	-26.75	0.267
Contrast	Zoomed	44.05, 35.81–69.32		
	Unzoomed	75.00, 61.80–116.65	70.25	**<0.001**
	BLI	42.13, 33.19–66.41	-4.36	**0.003**
	BLC	43.32, 31.18–68.00	-1.67	0.679
Correlation	Zoomed	0.98, 0.98–0.98		
	Unzoomed	0.97, 0.96–0.97	-1.28	**<0.001**
	BLI	0.98, 0.98–0.98	0.00	**0.022**
	BLC	0.98, 0.97–0.98	-0.15	0.349
Variance	Zoomed	1,299.83, 802.38–2,081.63		
	Unzoomed	1,312.13, 1,047.72–2,123.97	0.95	0.349
	BLI	1,342.30, 834.83–2,090.65	3.27	0.557
	BLC	1,276.80, 845.90–2,071.28	-1.77	0.396
IDM##	Zoomed	0.233, 0.182–0.315		
	Unzoomed	0.190, 0.148–0.258	-18.23	**<0.001**
	BLI	0.221, 0.186–0.291	-4.88	0.078
	BLC	0.249, 0.185–0.292	6.79	0.5
Sum average	Zoomed	90.36, 68.64–121.92		
	Unzoomed	91.86, 70.48–123.22	1.66	0.133
	BLI	93.99, 70.47–119.52	4.02	0.472
	BLC	89.22, 69.16–119.17	-1.26	0.472
Sum variance	Zoomed	5,164.32, 3,157.63–8,219.63		
	Unzoomed	5,181.82, 4,122.93–8,329.45	0.34	0.286
	BLI	5,336.16, 3,290.79–8,287.48	3.33	0.616
	BLC	5,072.63, 3,327.85–8,202.29	-1.78	0.372
Sum entropy	Zoomed	5.36, 5.04–5.57		
	Unzoomed	5.39, 5.08–5.56	0.53	0.679
	BLI	5.39, 5.15–5.65	0.51	**0.011**
	BLC	5.36, 5.04–5.64	0.08	0.586
Entropy	Zoomed	7.26, 6.72–7.70		
	Unzoomed	7.39, 6.94–7.86	1.79	**0.001**
	BLI	7.71, 7.13–8.00	6.14	**<0.001**
	BLC	7.64, 7.10–7.96	5.17	**<0.001**
Difference variance	Zoomed	22.13, 17.46–30.19		
	Unzoomed	37.02, 29.07–50.07	67.29	**<0.001**
	BLI	20.68, 15.95–28.00	-6.54	**0.002**
	BLC	21.41, 16.54–32.15	-3.23	0.248
Difference entropy	Zoomed	2.60, 2.47–2.81		
	Unzoomed	2.83, 2.72–3.05	9.05	**<0.001**
	BLI	2.57, 2.46–2.80	-1.04	**0.011**
	BLC	2.59, 2.44–2.81	-0.43	0.248
IMC-1*	Zoomed	-0.383, -0.406–0.358		
	Unzoomed	-0.352, -0.366–0.332	-7.94	**<0.001**
	BLI	-0.375, -0.411–0.356	-1.94	0.372
	BLC	-0.369, -0.402–0.352	-3.59	0.071
IMC-2**	Zoomed	0.981, 0.978–0.985		
	Unzoomed	0.977, 0.967–0.981	-0.46	**<0.001**
	BLI	0.985, 0.979–0.989	0.36	**<0.001**
	BLC	0.984, 0.977–0.987	0.23	0.306

# ASM: Angular second moment, ## IDM: Inverse difference moment, *IMC-1: Information measure of correlation-1, **IMC-2: Information measure of correlation-2

**Table 5:** Table showing the difference of GLDS features in zoomed images and those without zoom. The latter were subsequently resampled to equalise their resolution (pixels/mm) with the corresponding zoomed ones, by using bilinear (BLI) or bicubic (BCI) interpolation. Percent difference with zoomed image are also shown. Statistically significant differences are highlighted.

**GLDS features**	**Magnification**	**Feature value (median, interquartile range)**	**Difference with original (x1) image %**	**p**
Homogeneity	Zoomed	0.233, 0.182–0.315		
	Unzoomed	0.191, 0.149–0.258	-18.14	**<0.001**
	BLI	0.222, 0.186–0.291	-4.83	0.078
	BLC	0.249, 0.186–0.293	6.92	0.5
Contrast	Zoomed	43.87, 35.74–69.05		
	Unzoomed	74.70, 61.62–116.23	70.27	**<0.001**
	BLI	41.96, 33.11–66.14	-4.35	0.003
	BLC	43.22, 31.11–67.69	-1.49	0.679
Energy	Zoomed	0.092, 0.075–0.115		
	Unzoomed	0.074, 0.059–0.089	-19.79	**<0.001**
	BLI	0.095, 0.074–0.111	2.38	0.17
	BLC	0.095, 0.073–0.115	2.21	0.267
Entropy	Zoomed	2.63, 2.49–2.84		
	Unzoomed	2.86, 2.75–3.08	8.63	**<0.001**
	BLI	2.60, 2.49–2.84	-1.25	**0.012**
	BLC	2.61, 2.48–2.85	-0.65	0.306
Mean	Zoomed	4.75, 4.00–6.11		
	Unzoomed	6.24, 5.32–7.87	31.28	**<0.001**
	BLI	4.62, 3.97–5.98	-2.81	**0.003**
	BLC	4.65, 3.96–6.07	-2.10	0.845

**Table 6:** Table showing the difference of Fourier features in zoomed images and those without zoom. The latter were subsequently resampled to equalise their resolution (pixels/cm) with the corresponding zoomed ones, by using bilinear (BLI) or bicubic (BCI) interpolation. Percent difference with zoomed image are also shown. Statistically significant differences are highlighted.

**Fourier feature**	**Magnification**	**Feature value (median, interquartile range)**	**Difference with original (x1) image %**	**p**
Radial sum	Zoomed	2,881.53, 2,633.78–3,408.98		
	Unzoomed	2,316.44, 2,011.57–2,730.38	-19.61	**<0.001**
	BLI	3,006.34, 2,464.29–3,358.66	4.33	0.983
	BLC	2,818.77, 2,357.42–3,324.80	-2.18	0.679
Angular sum	Zoomed	2,547.76, 2,081.04–3,041.93		
	Unzoomed	1,883.20, 1,599.28–2,428.14	-26.08	**<0.001**
	BLI	2,406.81, 1,964.50–2,908.85	-5.53	0.231
	BLC	2,404.97, 1,995.90–2,667.91	-5.60	0.248

**Table 7:** Table showing the difference of Runlength features in zoomed images and those without zoom. The latter were subsequently resampled to equalise their resolution (pixels/cm) with the corresponding zoomed ones, by using bilinear (BLI) or bicubic (BCI) interpolation. Percent difference with zoomed image are also shown. Statistically significant differences are highlighted.

**Runlength feature**	**Magnification**	**Feature value (median, interquartile range)**	**Difference with original (x1) image %**	**p**
SRE	Zoomed	0.930, 0.914–0.950		
	Unzoomed	0.941, 0.923–0.958	1.14	**<0.001**
	BLI	0.944, 0.927–0.955	1.43	**<0.001**
	BLC	0.935, 0.918–0.951	0.48	**0.031**
LRE	Zoomed	1.52, 1.29–1.78		
	Unzoomed	1.37, 1.24–1.61	-9.82	**<0.001**
	BLI	1.36, 1.26–1.58	-10.47	**0.001**
	BLC	1.48, 1.28–1.72	-2.79	0.122
GLD	Zoomed	200.81, 116.00–284.64		
	Unzoomed	113.29, 68.86–223.80	-43.58	**<0.001**
	BLI	163.59, 97.78–265.25	-18.54	**0.02**
	BLC	202.02, 105.73–254.68	0.60	**0.008**
RLD	Zoomed	9,803.1, 7,905.6–17,216.9		
	Unzoomed	7,075.41, 5,557.61–9,965.58	-27.82	**<0.001**
	BLI	11,850.5, 9,793.2–17,921.7	20.89	0.078
	BLC	13,755.5, 10,062.6–17,620.4	40.32	**0.039**
RP	Zoomed	11.55, 9.25–19.41		
	Unzoomed	8.20, 6.28–11.88	-29.03	**<0.001**
	BLI	13.95, 10.80–20.76	20.79	0.157
	BLC	16.14, 11.82–20.20	39.79	0.078

On the other hand, most second order features (21/25, 84%) were significantly (p < 0.05) sensitive to the relatively small zoom factor of 1.3 (Table 4, 5, 6, 7). Compared with histogram features and first order statistics combined, second order statistics were significantly more often sensitive to zooming (p < 0.001, Fisher's exact test).

Resolution standardisation, indeed, decreased significantly these differences. This was more evident when the features, which were resolution sensitive, were considered separately (Table 8). The bicubic interpolation method was statistically significantly better than the bilinear interpolation method; this was more evident in the subgroup of features that are resolution dependent (Table 8), where the magnitude of change is on average 43% less for the 25 sensitive features (2.79% vs 4.88%).

**Table 8:** Image resampling reduces significantly the variability due to different image resolution. Bicubic interpolation (BCI) was better than bilinear interpolation (BLI), p = 0.036. This was remarkable for those 25 features shown on Wilcoxon analysis to be significantly different, when zoomed and un-zoomed images were compared. Results are shown as median and interquartile range.

	**Percent difference in comparison with unzoomed image**		
	All textural features (n = 50)	Significant textural features (n = 25)	Non significant textural features (n = 25)

Zoomed image (Z)	8.2%, 1.8–25.0	18.14%, 4.94–28.43	3.72%, 1.50–9.00
Resampled image (BLI)	4.5%, 2.7–12.0	4.88%, 1.75–11.49	4.31%, 3.24–12.43
Resampled image (BCI)	3.0%, 0.8–6.8	2.79%, 0.66–6.86	3.60%, 1.00–7.44
*Group comparison*			
Z vs BLI	0.112	0.004	0.26
Z vs BCI	0.011	0.006	0.43
BLI vs BCI	0.036	0.35	0.042

# IDM: Inverse difference moment, *IMC-1: Information measure of correlation-1, **IMC-2: Information measure of correlation-2

## Discussion

Our study showed that most second order textural features are particularly sensitive to the interpolation process during image zooming. Chan and McCarty reported that magnification affects runlength SRE, LRE and RP, but gave no further details [16]. This might be the result of increased pixel number. In contrast, most histogram features and first order statistics were relatively insensitive; actually these features are not texture algorithms.

A small zoom factor (the default by the ultrasound scanner was 1.3) is more likely to be applied in real circumstances, but under some circumstances this might be higher; the effect of a series of tests with progressively increased magnification could investigate if the association between zoom factor and change is linear, exponential, etc. It is expected that bigger zoom factors result in greater differences and further research is necessary to prove that this standardisation process eliminates any differences.

The implications of these results are that second order statistics should be used under standardised resolution settings, which means that these factors should be kept steady during the scanning process or a method of standardisation needs to be applied. In everyday practice, plaque resolution can vary up to 3 times, between 10–30 pixels/mm; this depends on the depth and zoom of the scanner. The former can vary from 2–5 cm. The combination of variable depth of carotid arteries and various zoom factors results in images of substantially different pixel number and therefore resolution (pixels/mm) of the region of interest (carotid plaque). This "normalised" resolution of the region of interest should not be confused with the image resolution, determined during the initial process of digitisation, for example all original images used in the current study hadresolution of 576 pixels (height) × 768 pixels (width). Increased depth results in reduced plaque resolution and although this can be controlled by zooming, so that resolution will remain the same, this is not possible in lengthy carotid plaques.

In the present study, two well-known interpolation methods were used to standardise resolution and it was found that the bicubic method is superior. This was expected, since the bicubic method is superior in terms of image quality, in comparison with the bilinear method [27,28]. The more complex algorithms, including bicubic interpolation, had the disadvantage of running slowly by the low-memory computers used in the 70^s ^and 80^s^, but modern technology has solved this problem. New algorithms, like the spline interpolation algorithm could be tested by future studies [29].

## Conclusion

Second order statistics, unlike most first order statistics and histogram features, are sensitive to image interpolation, commonly used during scanning with image zoom. A process of standardisation like the one used in this study should be applied when these features are used in images with variable resolution of the region of interest.

## Competing interests

The author(s) declare that they have no competing interests.

## Authors' contributions

SKK, ANN and GG designed the study. SKK conveyed the study and performed the statistical analysis. CSP and EK designed the image processing software used in the study. All authors helped to draft the manuscript and also read and approved the final manuscript.

## Appendix I

Bilinear interpolation algorithm

Bilinear interpolation determines the value of new pixels by calculating the weighted average of the values of the four surrounding pixels that is above, below, right, and left of the point where the new pixel is to be created (a 2 × 2 array).

Bicubic interpolation algorithm

Bicubic interpolation determines the values of new pixels by calculating the weighted average of the closest 16 pixels (a 4 × 4 array) based on distance. Although bicubic interpolation is slower and therefore requires more computational time, it produces a much smoother image than the bilinear technique and therefore it is considered superior; for this reason it is the default image-enlargement technique in the vast majority of image processing software.
